# Unsatisfying mitral valve repair? The “Loop method”: a lifebelt to grab

**DOI:** 10.1186/s13019-021-01649-x

**Published:** 2021-09-26

**Authors:** Claudia Loardi, Marco Zanobini

**Affiliations:** 1 Department of Cardiac Surgery, Tours University Hospital, 37044 Tours Cedex 9, France; 2 Department of Cardiac Surgery, Centro Cardiologico Monzino IRCCS, Università degli Studi di Milano, Milan, Italy

**Keywords:** Mitral regurgitation, Mitral valve repair, Artificial chords

## Abstract

Intra-operative mitral valve repair failure is a common condition in patients with complex myxomatous disease requiring aortic re-clamping and application of a fast and easy surgical technique to correct the residual imperfection. Herein we describe a reproducible method of artificial chord reconstruction which preserves the previous accomplished acts and allows for accurate chordal length measurement basing on the annuloplasty as the reference level.

## Introduction

Over the last few decades, a better understanding of the structure, function and pathology of the mitral valve (MV) improved the surgical results associated with degenerative MV repair, resulting in excellent long-term survival and freedom from reoperation [1]. Currently, several well-described repairing approaches for anterior and posterior leaflet prolapse are available, including both resectional and non-resectional (respect philosophy) techniques [2].

The respect paradigm for the reconstruction of the leaflet prolapse is mainly based on neochordal implantation procedure, whose critical point is represented by the assessment of artificial chordae proper length. Although various manoeuvres have been described to ensure safe and reproducible results [3], this step remains intuitive and based on personal experience [4]. Especially in case of intra-operative reconstruction failure for complex disease, the surgeon must choose the most appropriate tool among all the available strategies to correct the residual defect.

We report a simple method of neochordae placement and height adjustment using the prosthethic annulus as the reference point to correct a peri-operative detected unsatisfying MV repair.

## Technique

Following MV reconstruction performed with surgeon’s preferred technique and annuloplasty ring placement, valve competency is statically tested by injecting saline into the ventricle; if no residual regurgitation nor scallop prolapse or billowing is elucidated in association with a sufficient and harmonic length of coaptation throughout a whole symmetric coaptation line, the result is optimal and the atriotomy is closed. Nevertheless, one of these situations may exist, thus leading the surgeon to perform additional procedures in order to obtain a perfect continent valve.

The same scenario may occur after intra-operative transaesophageal echocardiographic control detecting an unsatisfying mitral repair requiring re-clamping and valve re-examination.

**Fig. 1:**
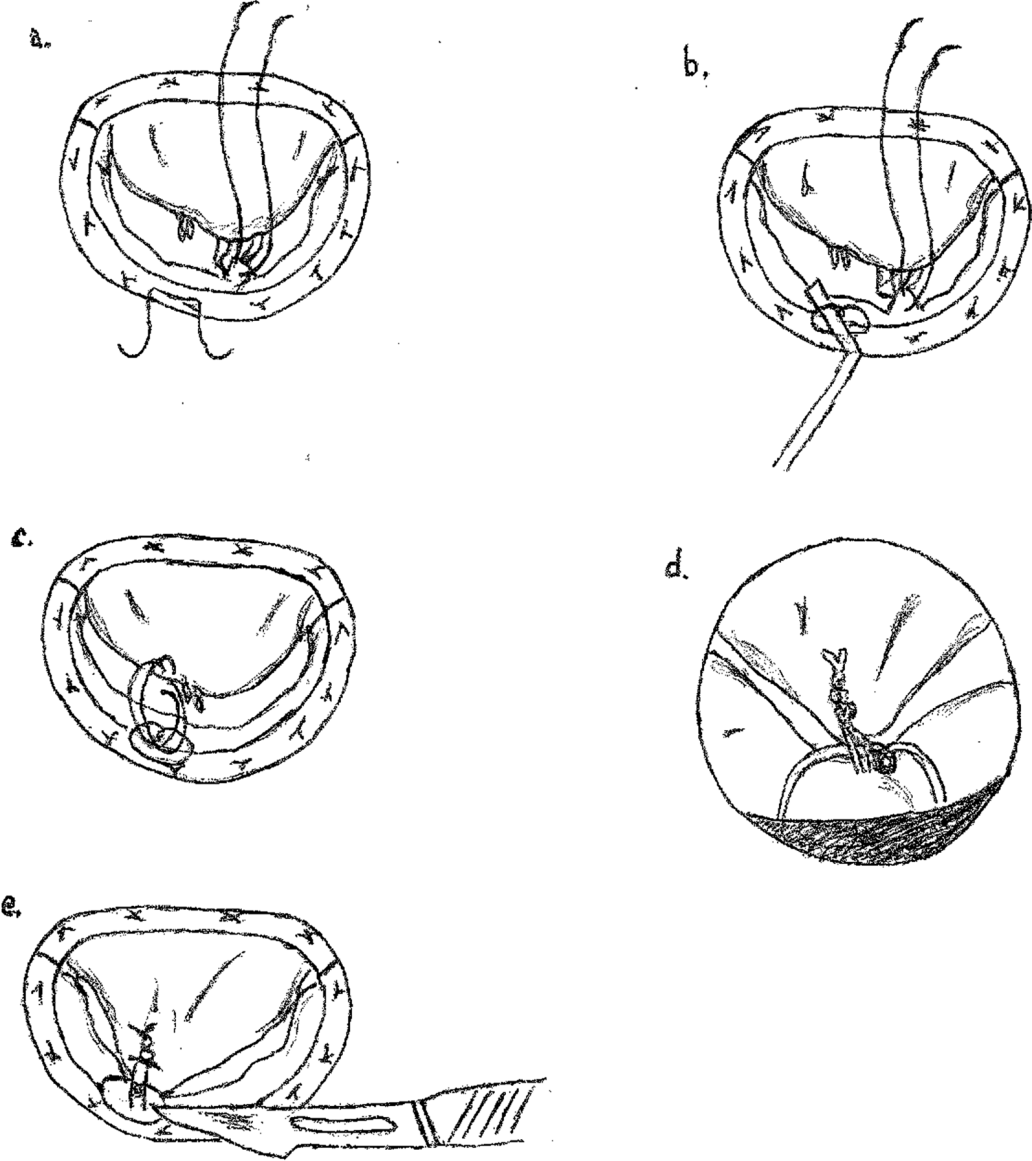
The “Loop method” technique for neochords length adjustment. **a** Placement of one or more Gore-Tex sutures in the papillary muscle and of an Ethibond stitch in the posterior part of the prosthethic ring. **b** The reference loop is created by tying the annular Ethibond suture leaning on a hook. **c** Both arms of the neochord are passed in the prolapsing area from the ventricular side to the atrial one, then in the loop from the atrium towards the ventricle and finally again into the edge of the prolapsing scallop from the ventricle to the atrium. **d** Knots are tied at the annular level. **e** The reference-loop is cut and the leaflet released

After detailed MV inspection, the prolapsing or poor coapting leaflets segments are easily identified. As a first step of the operative technique, one or more 4–0 Gore-Tex sutures (W.L. Gore & Associates, Flagstaff, AZ) are placed with a double passage in the appropriate papillary muscle head, oriented longitudinally and including the fibrous tip of the muscle (Fig. 1a). Papillary muscle exposure may be difficult in this phase due to the presence of the ring and of valvular repairing sutures: an unconnected aspirating cannula or a cylindrical mechanical prosthesis tester are helpful in reclining the anterior leaflet and facilitating the access to the subvalvular apparatus.

A 2/0 Ethibond Excel (Ethicon, Somerville, NJ) suture is passed in the posterior portion of the annuloplasty ring facing (in case of residual anterior leaflet prolapse) or at the basis (for the posterior one) of the guilty scallop and tied leaning on a surgical hook in order to form a loop representing the reference element for neochord length assessment (Fig. 1a, b). As the posterior ring is chosen as the seat of the guiding stitch, the technique is applicable in case of uncomplete annuloplasty too.

Now, both arms of the previously placed Gore-Tex suture are first passed through the margin of the prolapsing leaflet segment from the ventricular side to the atrial one, then through the reference-loop from the atrium to the ventricle and finally again into the edge of the prolapsing scallop from the ventricle to the atrium (Fig. 1c). Afterwards, knots are tied at the annular level (Fig. 1d) and the annular reference-loop is cut (Fig. 1e) to release the prolapsing leaflet.

Figure 1a–e describes the case of an anterior residual prolapse but the same procedure is equally applicable to a posterior one.

Such technique of artificial chordae height measurement can similarly be employed as the first approach for MV myxomatous disease repair by placing the loop stitch in the native annulus if the surgeon’s preference is to perform annuloplasty at the end of leaflet reconstruction or, on the contrary, in the prosthethic ring if the repairing sequence is neochordae positioning in the papillary muscle—annuloplasty—chords anchorage to the leaflet.

## Comment

Nowadays, MV repair includes a large spectrum of native pathologies (and consequently of surgical techniques) varying from the simple P2 prolapse until the Barlow disease with multi-scallop and commissural involvement [5]. In case of complex degenerative regurgitation, the first repairing attempt may result in an intra-operative (during the saline test or at transaesophageal echocardiographic check) unsatisfying result, consisting for instance in a residual limited prolapse or in an insufficient localized coaptation area, a recognized risk factor of long-term plasty failure [6]. Therefore surgeons are faced to the need of completing their previous act in a double difficult situation: a technical uncomfortable setting due to the pre-implanted cumbersome prosthethic ring and leaflet sutures associated to the requirement of performing a short and efficacious correction to limit cross-clamping time.

Our described technique (the “Loop method”) of artificial chord positioning solves both problems exploiting the presence of the annuloplasty ring and following the “respect philosophy”. Although alternative procedures can be employed in similar cases, such as an Alfieri stitch in the prolapsing area which surely represents a simpler solution, our preference is for Gore-Tex neochords since they avoid the danger of increasing trans-valvular gradient (in particular with a pre-existent annuloplasty sized in a different repairing situation) and allow a more physiologic repair.

Certainly, during the application of the technique in a “relooking” setting after a first attempt of mitral repair, great care must be addressed to avoid any interference or impingement between the last added element and the other chords (native or previously inserted). Moreover, as the surgical act may also modify MV set up and scallops interaction by incidentally opening artificial clefts or by slightly modifying the coaptation line, an accurate final check is essential.

Nevertheless, even if chordal replacement is recognized as a safe, effective and durable mitral reconstruction first line procedure, the well known critical challenge of determining the perfect chords height is topical once again and the codification of an easy height measurement method especially in this re-clamping setting is mandatory. More than 40 strategies are available in medical literature concerning such technical issue and are divided into two groups: those with premeasured required chordal length and those without [3]. Among those belonging to the last group, our “Loop method” presents some similarities with “The folding leaflet” one [7] but with the advantages of being independent from the assistant’s help and of permitting a certain knotting level at the prosthethic annular plane.

The existence of a plethora of techniques addressing the same issue suggests that none of them is reproducible in all hands, thus requiring the description of a standardized method very useful in case of primary plasty failure.

In conclusion, our technique is above all proposed as a simple and accurate “lifebelt” rescue strategy (even if it can be applied as a first procedure), whose main interest lies in its fast execution without the need of ring explantation and in a leaflet preserving approach.

## Data Availability

Not applicable.

## Abbreviation

MV: Mitral valve
